# Accuracy of MR arthrography in the detection of posterior glenoid labral injuries of the shoulder

**DOI:** 10.1007/s00256-022-04165-8

**Published:** 2022-08-25

**Authors:** Allison Rixey, Nicholas Rhodes, Naveen Murthy, Matthew Johnson, Nicholas Larson, Michael D. Ringler

**Affiliations:** 1 Department of Radiology, Mayo Clinic, Rochester, USA; 2 Department of Biomedical Statistics and Informatics, Mayo Clinic, Rochester, USA

**Keywords:** Posterior glenoid labrum, Posterior glenoid labral tear, MR arthrography, Shoulder arthroscopy

## Abstract

**Objective:**

The purpose of this study is to evaluate the accuracy of MR arthrography in detecting isolated posterior glenoid labral injuries using arthroscopy as the reference standard.

**Methods:**

MR arthrograms of 97 patients with isolated posterior glenoid labral tears by arthroscopy and those of 96 age and gender-matched controls with intact posterior labra were reviewed by two blinded radiologists for the presence and location of posterior labral abnormalities. The sensitivity and specificity of detection of posterior labral tears were calculated as well as the prevalence of associated pathologies. Medical records were reviewed for demographics, history and direction of shoulder instability, and prior surgery.

**Results:**

Posterior labral pathology was detected by MR arthrography with sensitivities of 76% and 84% for readers 1 and 2, and a specificity of 88% for both readers. Kappa value for interreader agreement was 0.91. Twenty-two of twenty-three (96%) tears isolated to the posteroinferior quadrant on arthroscopy were correctly identified on MRI. Commonly associated pathologies included paralabral cyst (38%), humeral fracture (7%), and glenoid fracture (2%). Fifteen of ninety-seven (16%) patients with posterior tears on both arthroscopy and MRI had glenoid rim deficiency on imaging versus no patients with intact posterior labra (*p* < 0.001). Forty of ninety-seven (41%) patients with posterior tears on arthroscopy had a history of posterior instability versus none without posterior tears. There was no significant difference in tear length on MRI between those with a history of instability and those without (*p* = 0.56).

**Conclusion:**

MR arthrography is accurate in detecting posterior glenoid labroligamentous injuries.

## Introduction

With its wide range of motion and relatively flat surface of the glenoid in relation to the humeral head, the glenohumeral joint is the most frequently dislocated joint in the body [1, 2]. Literature has described approximately 2–5% of glenohumeral dislocations and 10–24% of clinical instability as posterior in direction [3–6]. Athletes with overhead arm activity or in contact sports, patients in motor vehicle accidents, and patients with seizures are more prone to posterior instability and subluxation and/or dislocation events due to both macro and microinstability [1, 3, 7–9]. Due to their destabilizing effect on the posterior band of the inferior glenohumeral ligament and posteroinferior joint capsule, isolated posteroinferior labral tears predispose patients to recurrent instability and dislocation [2, 7, 10].

Clinically, posterior instability and labral pathology can be difficult to diagnose as patient-provided history and physical exam maneuvers can be nonconfirmatory [6, 10, 11]. Posterior glenoid labral pathology can manifest through a constellation of symptoms including pain, clicking, and shoulder fatigue with activity [5, 8, 10]. Given the high degree of recurrence, surgical management of these tears is often pursued early in the treatment course. Operative management is more successful when all components of instability are addressed, potentially involving labral repair, capsular shift and plication, and glenoid osteotomy with bone block procedures [6, 12, 13]. Posterior capsular shift and plication involve mobilization of redundant posterior glenohumeral joint capsular tissue followed by folding and securing the capsule and lax glenohumeral ligaments with suture anchors. During capsular plication, a radiofrequency probe can be used to stimulate capsular tissue and cause retraction, further tightening the glenohumeral joint. In patients with glenoid hypoplasia or retroversion, osteotomy and bone augmentation with iliac crest autograft or distal tibial allograft can correct for posterior glenoid deficiency to prevent subluxation or dislocation [6, 8, 12, 13]. Location of the pathology seen on MRI can affect portal placement during arthroscopy [14].

Given the difficulty of clinical diagnosis, differing surgical options, and recurrent nature of untreated injuries, accurate preoperative evaluation and localization are important. The purpose of this study is to assess the accuracy of direct MR arthrography in detecting posterior glenoid labral injuries using arthroscopy as the reference standard, as has been done for anterior and superior glenoid labral pathology.

## Methods

### Patient selection

Institutional review board approval was obtained for this retrospective, HIPAA-compliant study. The Enterprise’s electronic health records were initially screened based on structured and unstructured data elements for patients that had undergone shoulder arthroscopy between 2003 and 2018. Patients were considered eligible for analysis if they were 14 to 60 years of age at time of surgery and had an MR arthrogram within 100 days prior to surgery. Patients with prior ipsilateral shoulder surgery were excluded. The surgical database was then searched for patients that had undergone shoulder arthroscopy in which the posterior labrum was noted to be intact per the operative note. Age and gender-matched patients with MR arthrograms prior to surgery from this population were identified to be included in the control group. Patients with prior ipsilateral shoulder surgery were excluded. The electronic medical record was reviewed for patient demographics and for any history and direction of acute or chronic shoulder instability or dislocation event.

**Fig. 1:**
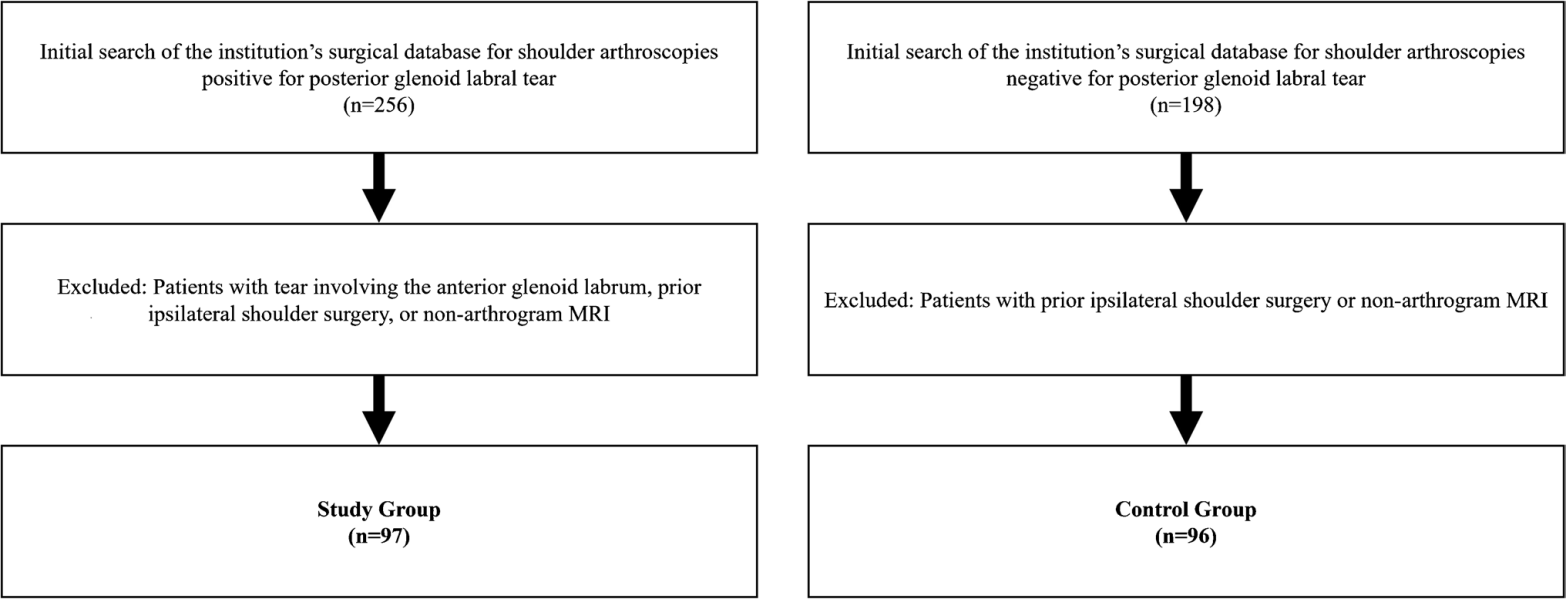
Inclusion and exclusion criteria for study and control groups

The initial search of the surgical database resulted in 256 patients with shoulder arthroscopy in which the operative note described a glenoid labral tear which affected the posterior labrum. Of this initial result, manual review of the clinical and operative notes from 11 different orthopedic surgeons consecutively identified 97 patients with tears isolated to the posterior labrum, had MR arthrography prior to surgery, and had no prior operative interventions to shoulder. The same surgical database was then searched for patients with intact posterior glenoid labra per operative note, until 96 patients with MR arthrography prior to surgery and no prior operative interventions to the shoulder were accrued (Fig. 1).

### Image acquisition

Following the intra-articular injection of a gadolinium-based contrast agent into the glenohumeral joint, imaging of the shoulder was obtained on multiple different MRI scanners at either 1.5 T or 3 T. A dedicated shoulder coil was used with the arm in neutral position. Most studies included the following sequences: axial and coronal turbo or fast spin echo fat-saturated T1-weighted images (TR/TE, 11–12/650–900; FOV, 140; slice thickness, 4 mm; slice interval, 0 mm; matrix 384 × 288–296, NEX 1, ETL 4), sagittal turbo or fast spin echo T1-weighted images (TR/TE, 11/650–900; FOV, 140; slice thickness, 4 mm; slice interval 0 mm, matrix 384 × 296, NEX 1, ETL 4), and axial, coronal, and sagittal turbo or fast spin echo fat saturated T2-weighted images (TR/TE, 45–49/4000; FOV, 140; slice thickness, 4 mm; slice interval 0 mm, matrix 388 × 288–296, NEX 1, ETL 10). Motion-degraded exams were not excluded.

### Image interpretation

Shoulder MR arthrograms from each subject were reviewed independently by two musculoskeletal fellowship-trained radiologists with 10 and 15 years of experience. The readers were blinded to age, gender, history of shoulder instability, prior imaging, MR arthrogram report, and arthroscopic findings. Examinations were evaluated for presence, location, morphology (classification), and length of glenoid labral tears. Location and length of tears were noted using a standard clockface [2]. Locations were further categorized into posteroinferior quadrant (6 o’clock to 9 o’clock) and/or posterosuperior quadrant (9 o’clock to 12 o’clock). A ± 1 h leeway was allowed for purposes of comparing MRI to arthroscopy report location to allow for reasonable interobserver differences, such that a tear confined to the “posteroinferior quadrant extending from 6 o’clock to 10 o’clock” in an arthroscopy report would be considered a match to a tear seen extending 6 o’clock to 9 o’clock on MRI or vice versa. Tears were classified in consensus using previously described eponymous/acronymous labral lesions, as a reverse Bankart lesion, posterior labrocapsular periosteal sleeve avulsion (POLPSA), posterior glenolabral articular disruption (PLAD), Kim lesion (superficial tearing between the posteroinferior labrum and glenoid articular cartilage), posterior humeral avulsion of the glenohumeral ligament (PHAGL), or unclassified [2]. Presence or absence of a paralabral cyst, humeral fracture (reverse Hill-Sachs), and glenoid fracture (bony Reverse Bankart) was also noted. Glenoid rim deficiency/dysplasia was graded based on findings on two contiguous axial slices as mild (rounding or truncation of the glenoid rim), moderate (rounding or truncation of the glenoid rim with some posterior tilt or sloping), or severe (extreme posterior tilt or sloping) as done previously by Harper et al. [15].

### Reference standard

Shoulder arthroscopy reports served as the reference standard for this study.

### Statistical analysis

Patient demographics and study characteristics were summarized as mean with standard deviation for continuous variables and total with percentage for categorical and ordinal variables. Using a binary categorization of tear present vs absent, diagnostic accuracy of the radiologists’ detection of posterior labral pathology was summarized by calculating sensitivity and specificity. Ninety-five percent confidence intervals for sensitivity and specificity were calculated using Wilson Score intervals. Inter rater agreement between the two readers for the binary diagnosis was assessed by Cohen’s kappa. All comparisons between groups used one-way ANOVA for continuous variables and chi-square test or Fishers exact test for categorical variables. *p*-values of < 0.05 were considered statistically significant throughout the analysis. All statistical analysis was performed using R version 4.1.2 (R Foundation for Statistical Computing, Vienna, Austria).

## Results

**Fig. 2:**
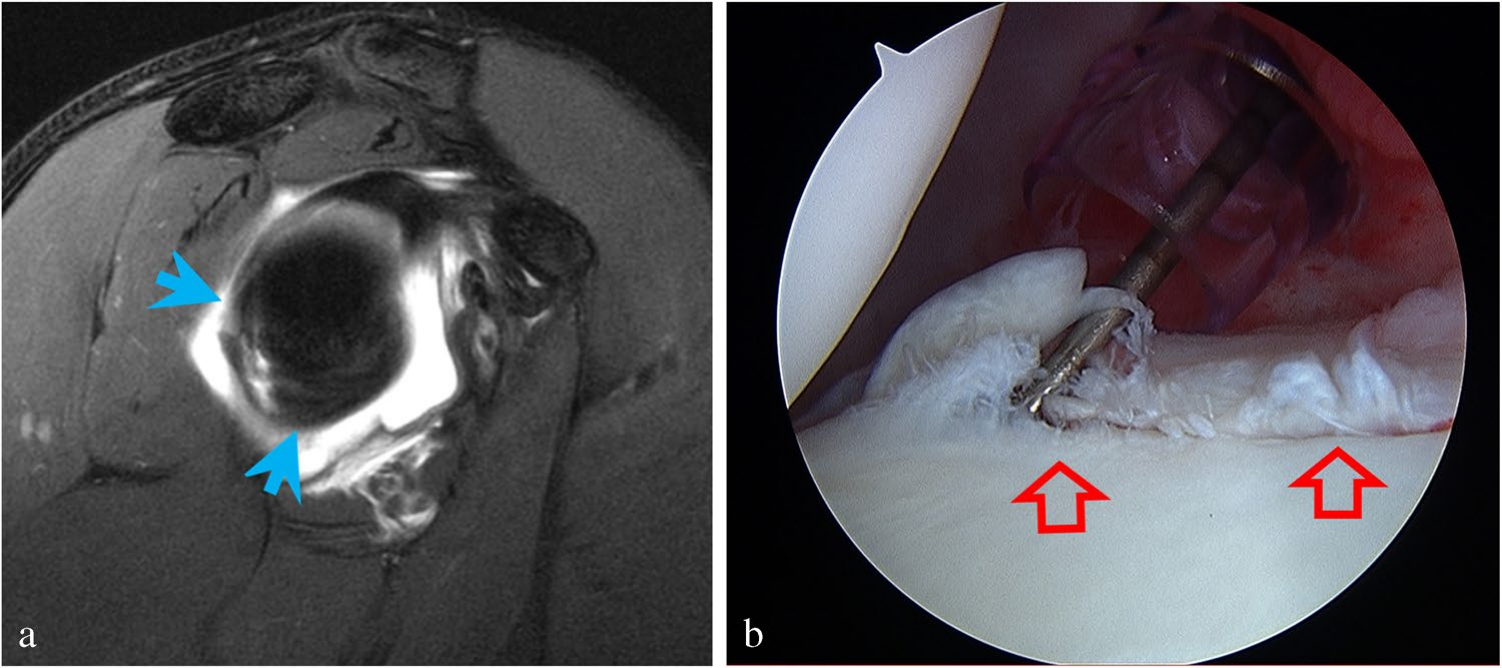
**a** Sagittal T2-weighted image with fat suppression shows a tear isolated to the posteroinferior glenoid labrum from the 6 o’clock to 9 o’clock position (blue arrows) in a 17-year-old male injured during football practice with acute onset posterior pain. **b** Arthroscopy confirmed a labral tear extending from the 6 o’clock to 10 o’clock position (red open arrows)

**Fig. 3:**
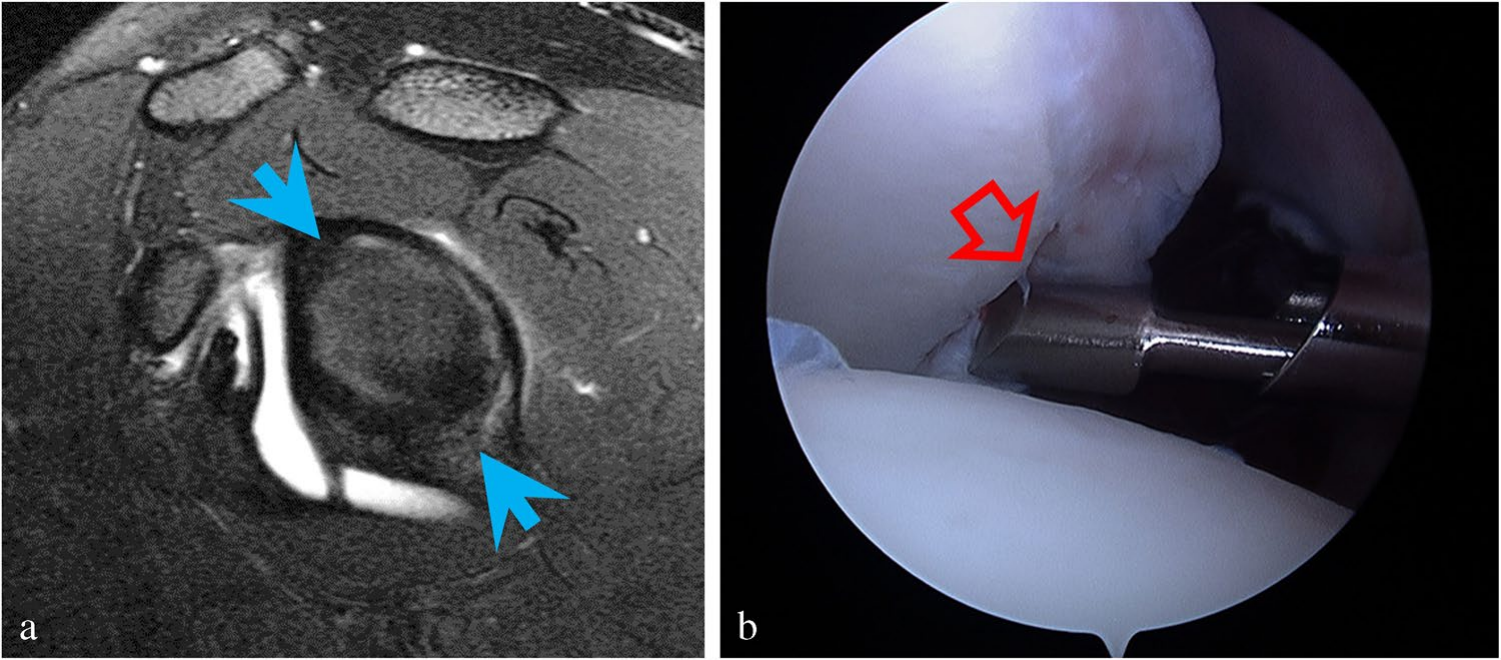
Nineteen-year-old male who sustained a posterior labral tear in the setting of a posterior glenhumeral dislocation playing football with recurrent subluxations. **a** Sagittal T2-weighted image with fat suppression shows a tear of the posterior labrum noted to extend between the 12 and 6 o’clock positions on the posterior labrum (blue arrows). **b** Arthroscopy correlation. The operative report noted posterior labral tear was confirmed from 7 o’clock to 9 o’clock, as seen lifted by the probe (red open arrow)

There was no significant difference in age (*p* = 0.80) or gender (*p* = 0.90) between the groups (Table 1). Posterior labral pathology was correctly detected by MR arthrography with sensitivities of 76% (95% CI = [67%, 84%]) and 84% (95% CI = [75%, 90%]) for readers 1 and 2, respectively, and a specificity of 88% (95% CI = [79%, 93%]) for both readers. The kappa value for interreader agreement was 0.91 (95% CI = [0.85, 0.97]). A total of 22/23 (96%) of labral tears isolated to the posteroinferior quadrant according to arthroscopy reports were also identified in the same quadrant on MRI (Fig. 2a-b). However, 6/22 (26%) of these cases were seen to also extend into the posterosuperior labrum beyond the 10 o’clock position on MRI (Fig. 3a-b). Twenty-three tears extended to the 12 o’clock position. The remaining cases all involved the posterosuperior quadrant.

**Table 1:** Age and gender comparison between study and control groups

	Study group (*n* = 97)	Control group (*n* = 96)	*p*-value
Gender			0.90
Male	86 (88.7%)	84 (87.5%)	
Female	11 (11.3%)	12 (12.5%)	
Mean age (range)	24.6 years (14–58)	25.5 years (14–54)	0.80

Of the 97 patients with posterior glenoid labral pathology on arthroscopy, 40 (41%) had a clinical history of isolated posterior shoulder instability. There was no significant difference in average length of labral tear on MRI measured on a clockface between those with a history of instability, measuring 3.6 h, and those without, measuring 3.3 h (*p* = 0.56).

**Fig. 4:**
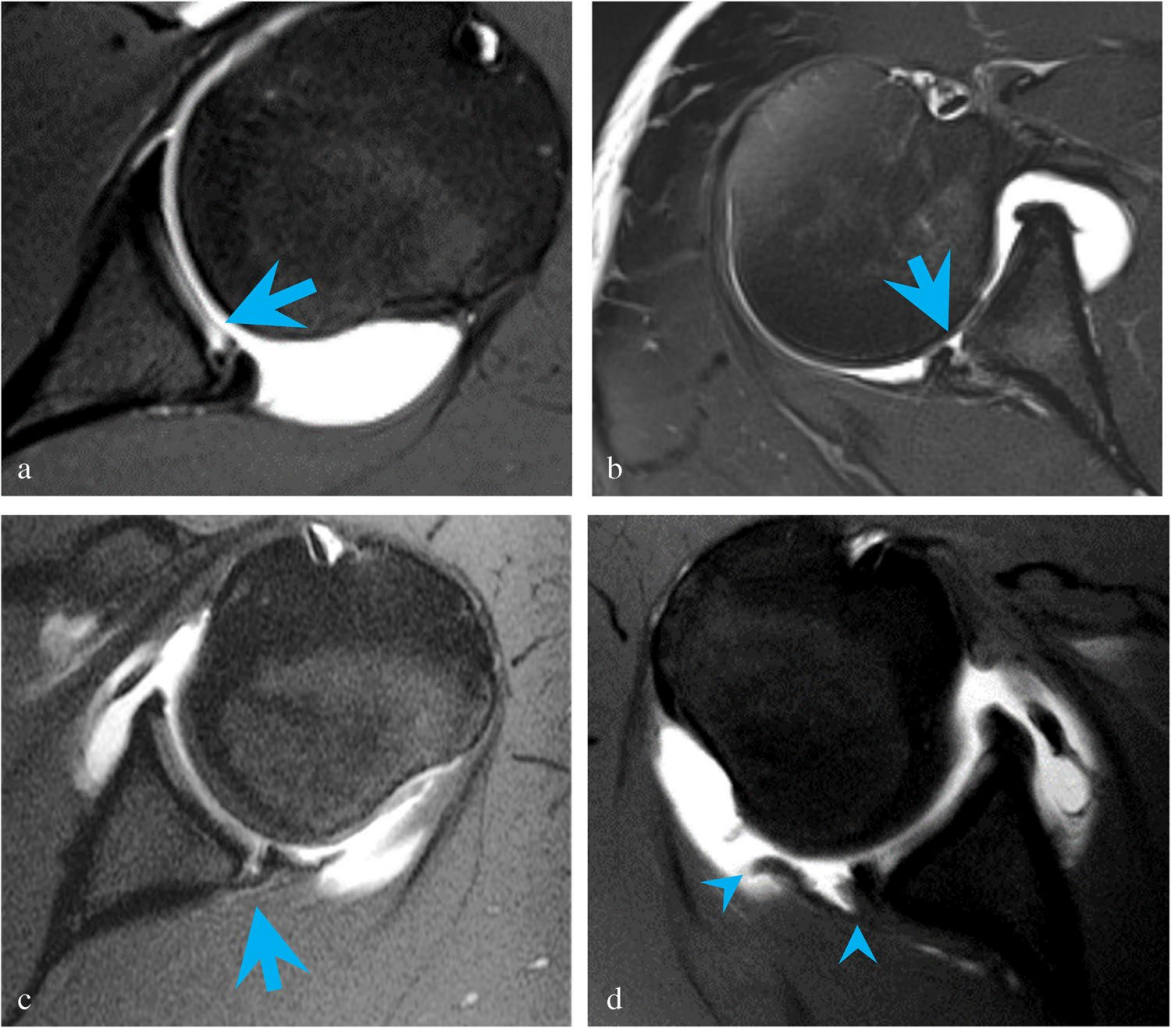
**a** Axial T1-weighted image with fat suppression demonstrates posterior glenolabral articular disruption (PLAD) with tear of the articular cartilage (blue arrow) in a 17-year-old male pitcher with 6 months of pain and instability following an acute subluxation event during batting. Arthroscopy showed a tear of the posterior labrum extending from 6-o’clock to 9-o’clock position with fraying of the posteroinferior glenoid cartilage. **b** Axial T2-weighted image with fat suppression shows a reverse Bankart lesion (blue arrow) in a 32-year-old male with pain and instability following a hockey injury. A slightly displaced bony fragment was found at surgery. The posterior labrum was noted to be attached to the bony fragment but torn away from the remaining glenoid. **c** Axial T1-weighted image with fat suppression shows a posterior labrocapsular periosteal sleeve avulsion (POLPSA) with stripping of the periosteum (blue arrow) in an 18-year-old male with 6 months of pain and recurrent subluxations following an acute dislocation event with self-reduction playing football. Arthroscopy confirmed a posterior labral tear extending from 7 o’clock to 9 o’clock. **d** Axial T1-weighted image with fat suppression demonstrates a posterior humeral avulsion of the glenohumeral ligament (PHAGL) (avulsed ligament delineated by blue arrowheads) in a 17-year-old male with 3 months of pain and recurrent subluxations following an acute posterior dislocation event while checking another player during a hockey game. Arthroscopy confirmed a tear from the 6 o’clock to 10 o’clock position. The posterior capsule and posterior band of the inferior glenohumeral ligament were found to be avulsed from the insertion on the humeral head

**Fig. 5:**
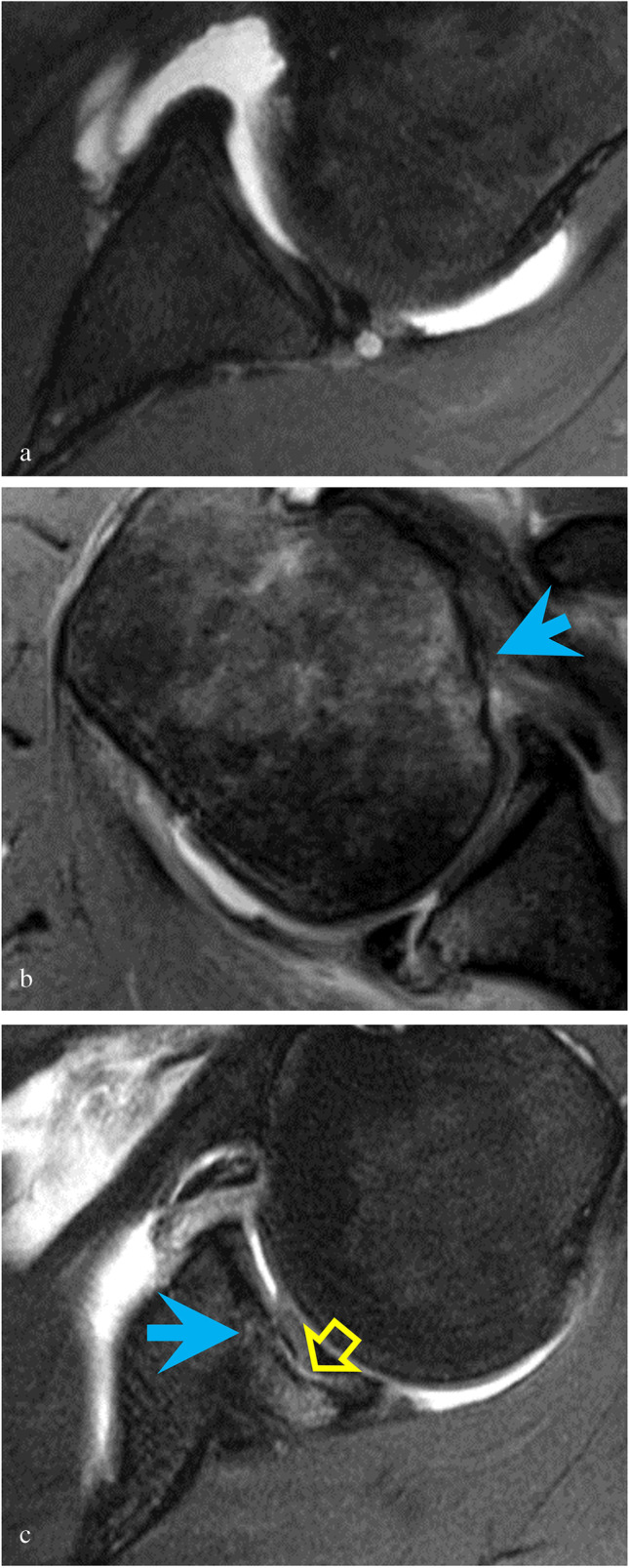
**a** Axial T2-weighted image with fat suppression shows a paralabral cyst in a 32-year-old male with an isolated posterior labral tear. **b** Axial T2-weighted image with fat suppression shows a humeral fracture (reverse Hill-Sachs) with cortical irregularity and flattening of the humeral head (blue arrow) in a 47-year-old male who suffered a posterior shoulder dislocation during a seizure and with 2 weeks of recurrent subluxations. **c** Axial T2-weighted image with fat suppression shows a glenoid fracture (reverse bony Bankart) with focal fracture and depression of the glenoid (blue arrow) and cartilage delamination (yellow open arrow) in a 31-year-old male former softball player who at the time of imaging unloaded heavy parcels at work. The osteochondral injury was noted to be unstable at surgery

Of the 83 correctly detected posterior labral tears, the majority (81%) did not fit a specific lesion type, while the remaining tears were classified as PLAD, Bankart, POLPSA, or PHAGL lesions (Table 2, Fig. 4a-d) by our radiologists. The most common associated pathology was paralabral cyst (38%), with humeral and glenoid fractures seen less frequently (Table 3, Fig. 5a-c). A total of 15/97 (16%) patients with posterior labral pathology on both arthroscopy and MRI were noted to have glenoid rim deficiency on imaging versus none (0/96) of the patients with intact posterior labra (*p* < 0.001).

**Table 2:** Lesion classification in posterior glenoid labral tears identified on both MR arthrography and arthroscopy

Lesion type	Total on arthrography (*n* = 83)
Unclassified	67 (81%)
Posterior glenolabral articular disruption (PLAD)	7 (8%)
Reverse Bankart	5 (6%)
Posterior labrocapsular periosteal sleeve avulsion (POLPSA)	3 (4%)
Posterior humeral avulsion of the glenohumeral ligament (PHAGL)	1 (1%)

**Table 3:** Pathologies associated with isolated posterior labral tears seen on MR arthrography

Associated pathology	Total
Paralabral cyst	36 (38%)
Humeral fracture (reverse Hill-Sachs)	7 (7%)
Glenoid fracture (reverse Bony Bankart)	2 (2%)

## Discussion

Our results indicate that MR arthrography is accurate in detecting posterior glenoid labral pathology and blinded reader evaluations showed high interreader agreement for evaluation of these injuries. An early study analyzing shoulder MRIs without intra-articular gadolinium showed the sensitivity and specificity for posterior labral tears to be 74% and 85%, respectively, in 19 patients [16]. A more recent meta-analysis of MR arthrography by Smith et al. calculated a sensitivity of 70% and specificity of 97% for posterior glenoid labral tears by pooling small numbers of cases from several studies [17]. While our results suggest that modern MR arthrography has a higher sensitivity of 76–84% for posterior labral tears than previous literature, this still remains slightly below the reported sensitivities for anteroinferior glenoid labral tears ranging between 88 and 92%, and SLAP tears between 84 and 98% [18–23]. Specificities for all labral lesions are comparable with previous reports ranging between 83 and 98% [18–23].

Consistent with the literature, most patients with posterior labral pathology identified in our study were young men [6]. Correlation with the medical record showed a higher incidence of posterior shoulder instability in patients with isolated posterior labral pathology, as expected. In patients without reported clinical instability, arthroscopy was most often pursued following failed conservative management for pain or return to sport or work. Contrary to one study, the length of labral tears in our series was not significantly associated with the clinical presence of posterior instability. Tung et al. reported a significant difference between patients with and without posterior instability using a tear length greater than 15 mm [24]. Glenoid size and morphology are known to vary considerably among different subjects with gender, height, and ethnicity contributing to height and width differences of up to 3–4 mm [25, 26]. Rather than absolute measurement, we chose to compare tear length using a clockface normalization, to account for these variations and found no such association. Despite having a larger number of patients, it is possible we did not have sufficient power to uncover such a relationship.

Most posterior labral tears seen in our cohort did not neatly fit into the list of archetypal eponymous or acronymous lesion types popular in review articles [2], and orthopedic surgeons almost never classified these tears in their operative reports. Thus, in practice, it is likely more helpful for radiologists to be descriptive in their reporting rather than focusing on naming the lesion, to allow for appropriate preoperative planning in this population which often suffers from recurrent symptoms [6, 8, 12]. Simply adding the words “reverse” or “posterior” to established anteroinferior labral injury types, in the setting of posterior labral tears, did not translate into added value in clinical management.

It is not surprising that patients with isolated posterior labral tears were significantly more likely to have glenoid rim deficiency, as many of these patients have a history of chronic instability and recurrent subluxations/dislocations. The literature has shown a significant correlation with glenoid dysplasia and symptomatic posterior shoulder instability which our study confirms [11, 15, 27]. It remains debatable if a deficient glenoid rim is secondary to recurrent subluxations/dislocations or if it is congenital and predisposes patients to posterior instability. Recognizing glenoid bone loss is imperative as its presence can predispose patients to failure of arthroscopic stabilization [4, 15].

Paralabral cyst was the most common associated pathology seen with labral tears. Humeral and glenoid fractures were relatively uncommon in our cohort which may be due to the relatively more common history of chronic instability rather than acute posterior dislocation.

There were limitations to this study, the first being the case–control, retrospective study design. Second, in reviewing the clinical notes, a clear history of shoulder instability or dislocation and the direction of shoulder instability were not always apparent based on subjective patient history or physical exam description. Additionally, inconsistencies in descriptions of arthroscopic findings among the 11 surgeons in the operative notes and the operator-dependent nature of arthroscopy may have resulted in inadvertent discrepancies in our reference standard. The operative note did not always specify in which quadrant the tear was located. Tears reported to be isolated to the posteroinferior quadrant on arthroscopy were not infrequently found to extend into the posterosuperior quadrant on MRI. This discrepancy may be the result of tears that have an intrasubstance component that is not unstable to probing during surgery or that have a component that healed prior to surgery. Another limitation is that MR arthrograms were obtained on several different scanners across the Enterprise, and protocols were not identical from scanner to scanner. Additional sequences available on some studies may have increased the radiologists’ confidence of diagnosing posterior labral pathology. However, the heterogeneity of exams and surgeons may make our results more widely applicable.

In summary, MR arthrography is accurate in detecting posterior glenoid labroligamentous pathology and associated abnormalities, and thus imaging is a reliable component in preoperative planning and management of these injuries.
